# Visualizing RNA Localization in *Xenopus* Oocytes

**DOI:** 10.3791/1704

**Published:** 2010-01-14

**Authors:** James A. Gagnon, Kimberly L. Mowry

**Affiliations:** Department of Molecular Biology, Cell Biology, and Biochemistry, Brown University

## Abstract

RNA localization is a conserved mechanism of establishing cell polarity.  Vg1 mRNA localizes to the vegetal pole of *Xenopus laevis* oocytes and acts to spatially restrict gene expression of Vg1 protein.  Tight control of Vg1 distribution in this manner is required for proper germ layer specification in the developing embryo.  RNA sequence elements in the 3' UTR of the mRNA, the Vg1 localization element (VLE) are required and sufficient to direct transport.  To study the recognition and transport of Vg1 mRNA *in vivo*, we have developed an imaging technique that allows extensive analysis of trans-factor directed transport mechanisms via a simple visual readout.

To visualize RNA localization, we synthesize fluorescently labeled VLE RNA and microinject this transcript into individual oocytes.  After oocyte culture to allow transport of the injected RNA, oocytes are fixed and dehydrated prior to imaging by confocal microscopy. Visualization of mRNA localization patterns provides a readout for monitoring the complete pathway of RNA transport and for identifying roles in directing RNA transport for cis-acting elements within the transcript and trans-acting factors that bind to the VLE (Lewis et al., 2008, Messitt et al., 2008).  We have extended this technique through co-localization with additional RNAs and proteins (Gagnon and Mowry, 2009, Messitt et al., 2008), and in combination with disruption of motor proteins and the cytoskeleton (Messitt et al., 2008) to probe mechanisms underlying mRNA localization.

**Figure:**
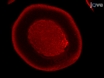


## Protocol

### Part 1: Transcription of Fluorescently Labeled mRNA.

1. Linearize plasmid DNA containing the RNA localization element or other relevant sequence and resuspend at 1 μg/μl with DEPC-treated H_2_O. The DNA template must have upstream promoter sites for transcription by T7, SP6, or T3 RNA polymerase.
2. Add the following reagents to a sterile 1.5mL tube:

**Table:** 

a. 10X Tx buffer (see M&M)	2 μl
b. 20X cap/NTP mix (see M&M)	1 μl
c. 1 mM Alexa Fluor 546-14-UTP (Invitrogen)	1 μl
d. UTP, [α-^32^P](1 μCi/μl) (Perkin Elmer)	1 μl
e. DEPC-H_2_O	11 μl
f. 0.2 M DTT	1 μl
g. RNasin (Promega)	1 μl
h. linear template DNA	1 μl
i. RNA Polymerase (Promega)	1 μl
3. Mix reagents gently and centrifuge briefly (10 sec. in a microcentrifuge).
4. Incubate for 2-4 hours at 37° C, covering the tube with aluminum foil to prevent photobleaching of fluorophore.
5. Add 1 μl 1 mg/ml RNase-free DNase (Promega) to degrade template DNA.
6. Incubate 15 minutes at 37° C.
7. Add 79 μl 20 mM EDTA (pH 8.0) to stop the reaction.
8. Remove 1 μl to a separate tube, labeled as "input" and save.
9. Spin reaction through a 1 ml G50 column (see Materials & Methods), which will include unincorporated nucleotides while excluding full length mRNA.
10. Concentrate the RNA by ethanol precipitation:
  1. Add 250 μl 100% ethanol, 10 μl 7M CH_3_COONH_4_, 1 μl carrier RNA (yeast tRNA, 10 μg/μl) or 1 μl glycogen (20 mg/ml).
  2. Mix well and freeze until solid at -80° C for >30 minutes or on dry ice for 15 minutes.
  3. Spin at maximum speed in microcentrifuge for 15 minutes, decant supernatant.
  4. Wash with 150 μl of 75% ethanol.
  5. Spin for 3 minutes at maximum speed in microcentrifuge, decant supernatant.
  6. Dry the pellet at 37° C for 3 minutes, ensuring that all ethanol has evaporated.
11. Resuspend in 11 μl DEPC-H_2_O and remove 1 μl to a separate tube, labeled as "incorporated".
12. Determine the percent incorporation and dilute the RNA to 50 nM (see Materials & Methods for calculation).
13. The RNA should be frozen in 5 μl aliquots, for single use to avoid freeze / thaw cycles and can be stored for several weeks at -80° C.

### Part 2: Preparing for Microinjection.

1. RNA Preparation:Thaw an aliquot of RNA (50 nM), and denature the RNA at 70° C for 3-5 minutes. Spin for 10 minutes in room temperature microcentrifuge at maximum speed to remove any particulates, and keep on ice.
2. Oocyte Preparation:
  1. Surgically remove ovary from *Xenopus laevis* females (Nasco).
  2. Trim pieces of *Xenopus laevis* ovary into a 50 ml conical tube containing 25 ml of Collagenase solution (see Materials & Methods).
  3. Shake gently at 18° C for 15 minutes, or until oocytes are visibly separated from ovary. Due to batch to batch variation, collagenase treatment time may vary and should be monitored carefully through visual inspection to ensure defoliculation.
  4. Allow oocytes to settle in tube, remove solution and wash the oocytes with MBSH buffer (see Materials & Methods).
  5. Repeat wash twice more for a total of three washes. This oocyte isolation protocol is similar to that detailed in Cohen et al., 2009.
  6. Manually sort stage III/IV oocytes (Dumont, 1972) in MBSH buffer under a standard dissecting microscope. Stage III oocytes are completely opaque but white, while stage IV oocytes are slightly larger and speckled with pigment. Oocytes that are slightly transparent are too young and oocytes that fully pigmented or exhibit polarized pigment distribution are too old.
3. Needle Preparation:Pull and bevel needles to an outer diameter of ~ 0.05 mm. (We use Drummond Scientific 3.5 inch capillaries (order # 3-000-203-G/X), a Sutter Instrument Co. micropipette puller and a WPI Inc. beveller.)

### Part 3: Microinjection

1. Calibrate needle with DEPC-H_2_O to deliver 2 nl per injection. We front load our needle using a gas-driven microinjector (see Materials & Methods), and calibrate drop size using a micrometer.
2. Load RNA into needle.
3. Place sorted oocytes into an injection dish containing MBSH buffer. We microinject on a dish with a layer of black foam rubber. The pale oocytes stand out well on a white background.
4. Carefully inject each oocyte with 2 nl of RNA at 50 nM.
5. Expel RNA, rinse needle with DEPC-H_2_O, and load next RNA for injection.

### Part 4: Oocyte Culture

1. Place oocytes in a well of a sterile 24 well plate (Sigma Aldrich). We culture up to a thousand oocytes per well.
2. Remove buffer and replace with 400 μl Oocyte Culture Medium (OCM; see Materials & Methods) per well. Place culture plate inside an air-tight plastic container with wet paper towel to maintain moist environment during culture.
3. Incubate oocytes at 18° C for time points ranging between 8 and 48 hours.

### Part 5: Fixation, Dehydration and Storage of Oocytes

1. Sort out any dead oocytes and place surviving oocytes in glass vials. We routinely observe >90% survival.
2. Place oocytes in MEMFA fixative (see Materials & Methods) and rock for 20 minutes. Protect the oocytes from light by covering with aluminum foil.
3. Wash oocytes by removing fixative and replacing with equal volume of MBSH buffer. Repeat this wash once more for a total of two washes.
4. Wash oocytes into anhydrous methanol:
  1. Remove half of the volume, replace with methanol.
  2. Repeat step "a" twice.
  3. Remove all of the solution, replace with methanol.
  4. Repeat methanol wash.
5. Oocytes can be stored at -20° C until ready to image.

### Part 6: Imaging RNA Localization by Confocal Microscopy

1. Before imaging, add Murray s Clearing Medium (2:1 benzyl benzoate-benzyl alcohol) to a glass bottom FluoroDish (WPI Inc.) to cover the imaging surface.
2. Carefully transfer the oocytes from methanol to FluoroDish, minimizing the volume of methanol transferred.
3. Image on an inverted confocal microscope (We have successfully used a Zeiss LSM510 and a Leica TCS SP2).

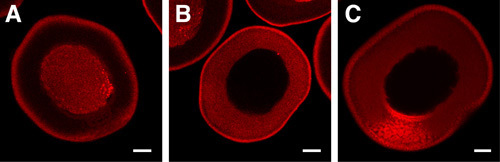
 **Figure 1: **Visualization of Subcellular RNA Localization by Microinjection of Fluorescently Labelled Transcripts.  **A.**  Following microinjection, RNA labeled with Alexa Fluor 546 is initially restricted to the nucleus. **B.** After eight hours of culture, a fluorescently labeled control RNA can be seen uniformly distributed in the cytoplasm of the oocyte. **C.** However, fluorescently labeled RNA containing sequences that recruit the transport machinery can be seen in the process of subcellular localization to the vegetal pole of the oocyte (towards bottom). Scale bar = 50 μm.

## Discussion

Here we have presented a protocol for visualizing mRNA localization in *Xenopus* oocytes. This method, using fluorescently labeled RNA transcripts has higher signal to noise ratio than previously obtained with digoxigenin-labeled transcripts and is simpler and faster than in-situ based approaches (Mowry and Melton, 1992, Gautreau et al., 1997). Using this method, we can engineer RNA sequence mutations and rapidly test for *in vivo* function. Further, by using additional Alexa-UTP fluorophores, multiple RNA species can be visualized in the same oocyte. We have found that in *Xenopus* oocytes Alexa Fluor 546-14-UTP gives superior results as compared with Alexa Fluor 488-5-UTP because of autofluorescence in the 488 nm wavelength range; however, dual imaging of two RNAs is nonetheless possible (Gagnon and Mowry, 2009). Also, this technique works well in conjunction with immunostaining protocols to detect protein colocalization, as a wide variety of compatible fluorescent secondary antibodies are available (Yoon and Mowry, 2006, Messitt et al., 2008). Finally, disruption of the RNA localization process through over-expression of dominant negative factors or small molecule interference can be combined with this assay to visualize subtle defects in the mRNA localization pathway (Messitt et al., 2008). This technique has proven to be a relatively simple and robust assay for detecting mRNA distribution in vivo and can be integrated easily into existing biochemical, molecular and cell biological techniques for probing mechanisms of mRNA transport.
